# Strain‐Induced Decoupling Drives Gold‐Assisted Exfoliation of Large‐Area Monolayer 2D Crystals

**DOI:** 10.1002/adma.202419184

**Published:** 2025-02-19

**Authors:** Jakob Ziewer, Abyay Ghosh, Michaela Hanušová, Luka Pirker, Otakar Frank, Matěj Velický, Myrta Grüning, Fumin Huang

**Affiliations:** ^1^ Centre for Quantum Materials and Technologies School of Mathematics and Physics Queen's University Belfast University Road Belfast BT7 1NN UK; ^2^ J. Heyrovský Institute of Physical Chemistry Czech Academy of Sciences Dolejškova 2155/3 Prague 18223 Czech Republic; ^3^ Faculty of Chemical Engineering University of Chemistry and Technology Prague Technická 5 Prague 6 16628 Czech Republic

**Keywords:** 2D materials, decoupling, gold‐assisted exfoliation, MoS_2_, Raman spectroscopy, strain

## Abstract

Gold‐assisted exfoliation (GAE) is a groundbreaking mechanical exfoliation technique that produces centimeter‐scale single‐crystal monolayers of 2D materials. Such large, high‐quality films offer unparalleled advantages over the micron‐sized flakes typically produced by conventional exfoliation techniques, significantly accelerating the research and technological advancements in the field of 2D materials. Despite its wide applications, the fundamental mechanism of GAE remains poorly understood. In this study, using MoS₂ on Au as a model system, ultra‐low frequency Raman spectroscopy is employed to elucidate how the interlayer interactions within MoS_2_ crystals are impacted by the gold substrate. The results reveal that the coupling at the first MoS_2_‐MoS_2_ interface between the adhered layer on the gold substrate and the adjacent layer is substantially weakened, with the binding force being reduced to nearly zero. This renders the first interface the weakest point in the system, thereby the crystal preferentially cleaves at this junction, generating large‐area monolayers with sizes comparable to the parent crystal. Biaxial strain in the adhered layer, induced by the gold substrate, is identified as the driving factor for the decoupling effect. The strain‐induced decoupling effect is established as the primary mechanism of GAE, which can also play a significant role in general mechanical exfoliations.

## Graphical abstract

Au substrate induces a strain in the adhered bottommost MoS_2_ layer. The strain weakens the coupling at the first MoS_2_‐MoS_2_ interface, making it the weakest point in the system, therefore MoS_2_ crystal preferentially cleaves at this interface, facilitating the selective exfoliation of large‐area monolayers with size comparable to that of the parent crystal.

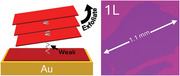

## 1 Introduction

Gold‐assisted exfoliation (GAE) is a groundbreaking mechanical exfoliation technique capable of producing centimeter‐scale single‐crystal monolayers of a diverse range of 2D materials.^[^ ^1^, ^2^, ^3^, ^4^ ^]^ It expands the exfoliated film area by several orders of magnitude compared to the conventional technique using scotch tape and SiO_2_ substrates.^[^ ^5^ ^]^ Although bottom‐up synthesis methods, such as physical vapor deposition and chemical vapor deposition, can yield wafer‐scale monolayers,^[^ ^6^, ^7^, ^8^ ^]^ their quality is often compromised by defects and polycrystallinity.^[^ ^9^, ^10^ ^]^ By contrast, GAE produces pristine monolayer crystals of superior quality.^[^ ^11^ ^]^ Combining scalability and excellent material quality, GAE provides unprecedented opportunities for advanced research and technological development in the field of 2D materials, becoming central to the construction of a wide variety of structures, including free‐standing monolayers, heterostructures, and moiré superlattices.^[^ ^12^, ^13^, ^14^, ^15^ ^]^

The GAE technique is broadly applicable to 2D materials containing sulfur (S), selenium (Se), or tellurium (Te), such as transition metal dichalcogenides (TMDCs) including MoS_2_, WS_2_, MoSe_2_, WSe_2_, MoTe_2_, WTe_2_, and various other chalcogenides like InSe, GaSe, In_2_Se_3_, Cr_2_Ge_2_Te_6_, and Fe_3_GeTe_2_.^[^ ^1^, ^2^ ^]^ It has also been applied to a few non‐chalcogenide 2D materials, such as graphene, hexagonal boron nitride (hBN), black phosphorus, CrSBr, and CrCl_3_.^[^ ^2^, ^13^, ^16^, ^17^ ^]^ To date, more than fifty layered materials have been successfully exfoliated with GAE.^[^ ^2^, ^18^ ^]^ Recently, the technique has been extended to alternative metals including Ag, Pd, Cu, Ni, and Co.^[^ ^19^, ^20^, ^21^ ^]^

Despite its extensive use and significant impacts in the field of 2D materials, the fundamental mechanism of GAE and, more generally, metal‐assisted exfoliation, remains poorly understood. Adhesion between 2D crystals and the gold substrate has been proposed as a key factor.^[^ ^1^, ^2^, ^22^ ^]^ It is assumed for the large‐area exfoliation to take place, the adhesion of the 2D crystal to the substrate needs to exceed the interlayer van der Waals (vdW) force within the 2D crystal.^[^ ^1^, ^2^ ^]^ However, this fails to explain why GAE predominantly produces large‐area monolayers with near‐unity yield, instead of randomly generating flakes of various thicknesses as is the case for the scotch tape/SiO_2_ method. The substrate‐induced strain has been hypothesized as a potential driver, weakening the coupling at the first interface between the adhered layer and the adjacent layer, favoring monolayer exfoliation.^[^ ^3^, ^22^, ^23^, ^24^ ^]^ However, these are only speculations. To date, solid experimental evidence supporting this hypothesis has been lacking, leaving critical aspects of the mechanism unresolved.

In this work, we present compelling experimental evidence to reveal the fundamental mechanism of GAE. Using MoS_2_ on gold as a model system,^[^ ^1^, ^2^, ^3^ ^]^ we characterized the force constants at the first MoS_2_‐MoS_2_ interface, i.e., the interface between the bottommost layer adhered to the gold substrate and the adjacent upper layer, through ultra‐low frequency (ULF) Raman spectroscopy.^[^ ^25^, ^26^, ^27^, ^28^ ^]^ Our findings reveal that the coupling at the first interface is significantly weakened during the exfoliation process, with the degree of decoupling depending on crystal thickness. For bilayers, the coupling is weakened by ≈20%, which increases to ≈50% for tetralayers, and ≈100% for crystals thicker than five layers. These results indicate that the first interface is the weakest point in the system. Therefore, MoS_2_ crystals preferentially cleave at this interface, generating large‐area monolayers on the gold substrate.

This finding is further supported by the observation of micron‐sized bubbles in annealed samples of MoS_2_ on Au. When samples of MoS_2_ on Au were annealed at 200 °C, micron‐sized bubbles were observed forming in flakes thicker than three layers but not in thin flakes of 1–3 layers. When the samples were further annealed at an elevated temperature of 400 °C, the bubbles burst, and all the exposed surfaces were shown to be monolayers. This provides direct experimental evidence unambiguously confirming that the first MoS_2_‐MoS_2_ interface is the weakest point in the system and that MoS_2_ crystals preferentially break at this interface. Biaxial strain in the adhered layer, induced by the gold substrate, is identified to be the primary driver of the decoupling effect. Density functional theory (DFT) simulations reveal that when the strain in the adhered layer and the crystal thickness exceed specific thresholds, monolayer exfoliation becomes energetically favorable.

## 2 Results and Discussions

### 2.1 Sample Fabrication and Thickness Characterization

**Figure 1:**
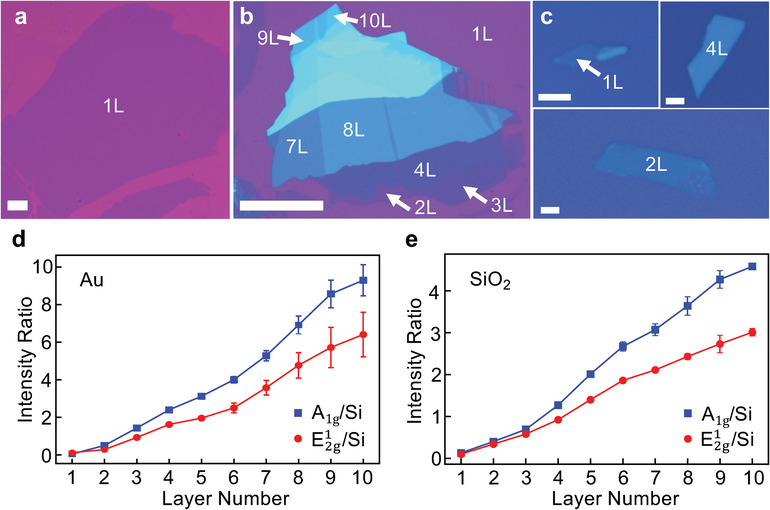
Optical images of MoS_2_ and thickness characterization. Optical images of large‐area monolayer a) and few‐layer b) MoS_2_ exfoliated on a 10 nm Au substrate. Scale bars: 100 µm. c) Optical images of MoS_2_ exfoliated on a SiO_2_(100 nm)/Si substrate. All scale bars are 2 µm. d,e) Raman intensity ratios of the MoS_2_ $E_{2g}^{1}$ and *A*_1g_ peaks to the Si peak at 520.7 cm^−1^ for 1–10L MoS_2_ on a 10 nm Au substrate (d) and a SiO_2_(100 nm)/Si substrate (e).

The processes of GAE have been described in a previous study,^[^ ^1^ ^]^ briefly summarized here: MoS_2_ crystals are cleaved just prior to exfoliation to minimize contamination; the cleaved surface is then pressed onto a freshly prepared Au substrate and lifted off, resulting in large‐area monolayers adhered to the Au substrate. A range of samples were prepared on thin Au films of various thicknesses (2, 5, and 10 nm) as well as on commercially purchased template‐stripped (TS) Au films of 100 nm. All thin Au films (unless otherwise stated) were deposited on a SiO_2_(100 nm)/Si substrate (referred to as SiO_2_ hereafter) with a 1 nm Ti adhesion layer through magnetron sputtering. For all Au samples, the exfoliated MoS_2_ crystals are predominantly monolayers exceeding 1 mm in lateral dimension (limited by parent crystal size), as shown in **Figure** **1**a and Figure S1 (Supporting Information). Besides the monolayers, there are usually some scarcely distributed multilayers (Figure 1b). For comparison, we also prepared MoS_2_ crystals on bare SiO_2_ substrates as reference samples, which comprise small flakes (<20 um) of various thicknesses (Figure 1c). More details of sample fabrication are described in Methods and in previous publications.^[^ ^1^, ^29^ ^]^

The thickness of MoS_2_ layers is determined through multiple characterizations, including optical contrast, Raman, and AFM measurements. For example, the intensity ratios between the Raman modes ($E_{2g}^{1}$ and *A*_1g_ modes, using the bulk notation) of MoS_2_ and the underlying Si substrate (520.7 cm^−1^ peak) are shown to be quasi‐linearly proportional to the layer number (Figure 1d,e), which can be used to accurately determine the thickness of MoS_2_.^[^ ^30^ ^]^ This, corroborated with optical contrast, ULF Raman, and AFM measurements (Figure S2, Supporting Information), allows us to accurately identify 1–10 layer MoS_2_ on Au and SiO_2_.

### 2.2 High‐Frequency Raman Spectra: Strain in the Adhered Layer

**Figure 2:**
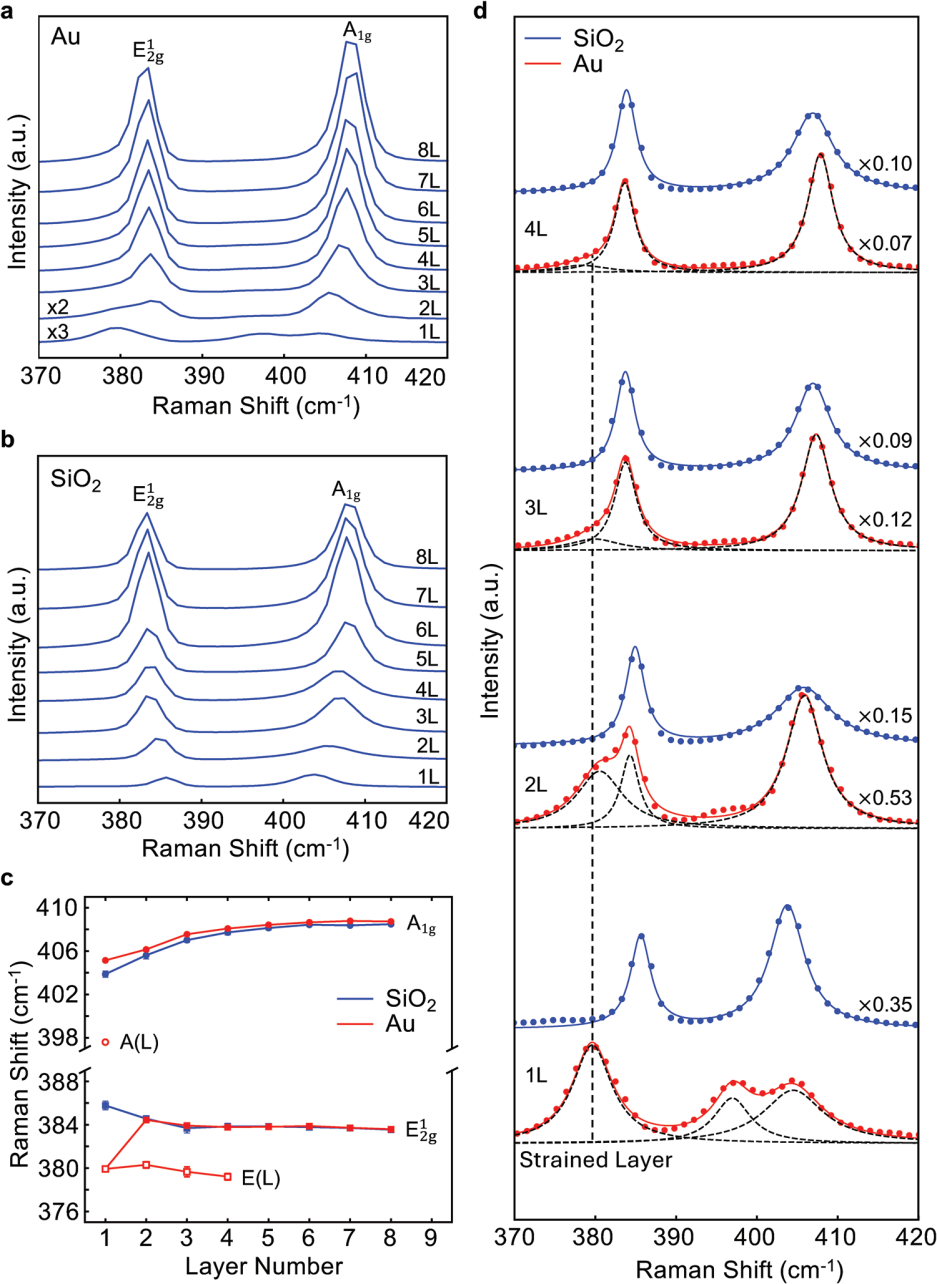
High‐frequency Raman spectra of MoS_2_. Raman spectra of 1–8L MoS_2_ on a) 10 nm Au and b) SiO_2_ substrates. c) Peak frequencies of the Raman modes. The $E_{2g}^{1}$ modes of 2–4L MoS_2_ on Au consist of two peaks, a low‐frequency peak E(L) ≈380 cm^−1^, close to the $E_{2g}^{1}$ mode of the monolayer on Au, and a high‐frequency peak matching that on SiO_2_, suggesting that E(L) originates from the strained bottom layer, and the high‐frequency peak originates from the top unstrained layer(s). d) Comparison of the Raman spectra of 1–4L MoS_2_ on SiO_2_ and Au substrates. Dashed lines: Lorentzian fitting of Raman peaks. The vertical line marks the peak position of the $E_{2g}^{1}$ mode of monolayer on Au. Scaling factors are normalized to the spectrum of monolayer on Au.

We first investigated the high‐frequency Raman spectra of MoS_2_. **Figure** **2** shows examples of the high‐frequency Raman spectra of MoS_2_ on 10 nm Au (Figure 2a) and SiO_2_ (Figure 2b) substrates. The high‐frequency Raman spectra show two main vibration modes, $E_{2g}^{1}$ mode (in‐plane) and *A*_1g_ mode (out‐of‐plane). The frequency and intensity of both modes are thickness‐dependent. For MoS_2_ films on SiO_2_, both the $E_{2g}^{1}$ mode and the *A*_1g_ mode displays a single peak. The *A*_1g_ mode upshifts while the $E_{2g}^{1}$ mode downshifts with increasing thickness, in agreement with prior literature.^[^ ^31^ ^]^

For MoS_2_ on Au, the spectra are notably different. The $E_{2g}^{1}$ mode of a monolayer displays a single peak at 380 cm^−1^ (Figure 2d), which is downshifted by 6 cm^−1^ with respect to its SiO_2_ counterpart (386 cm^−1^). The downshift of the $E_{2g}^{1}$ mode indicates the presence of a biaxial strain in the monolayer.^[^ ^29^, ^32^ ^]^ Based on the frequency shift, the mean strain is estimated to be 1.1% using a literature‐averaged Grüneisen parameters of 0.71 (using the formula $\varepsilon =\frac{\Delta \omega }{2\gamma \omega_{0}}$, Δ*ω* is frequency shift, *ω*_0_ is the frequency at zero strain, and *γ* is the Grüneisen parameter),^[^ ^32^, ^33^, ^34^ ^]^ close to the 1.2% reported previously.^[^ ^29^ ^]^

The $E_{2g}^{1}$ mode of 2–4L MoS_2_ on Au shows splitting, which can be fitted with two Lorentzian peaks (Figure 2d). The peak positions of Raman modes averaged from several spectra are presented in Figure 2c. As seen from Figure 2c,d, the low‐frequency peaks of the MoS_2_ $E_{2g}^{1}$ doublets on Au (denoted as E(L) in Figure 2c) remain at ≈380 cm^−1^ for the bilayer, trilayer, and tetralayer, closely matching that of the monolayer. This suggests that the E(L) peak originates from the bottom strained layer adhered to the Au substrate. On the other hand, the high‐frequency components of the $E_{2g}^{1}$ doublets match those on SiO_2_, implying that they originate from the top layer(s), which are little affected by the strained bottom layer. This indicates that the biaxial strain is mostly localized in the bottom adhered layer with little transfer to subsequent layers, consistent with theoretical simulations.^[^ ^24^, ^35^ ^]^ These results are observed on all Au samples, with some variance in the shift of the monolayer E(L) peaks, with the lowest being (377.8 ± 0.1) cm^−1^ for 2 nm Au samples and the highest being (380.0 ± 0.1) cm^−1^ for 5 nm Au samples, indicating a strain ranging from roughly 1.5% to 1.1%.

Unlike the $E_{2g}^{1}$ mode, the *A*_1g_ mode of monolayer MoS_2_ on Au shows a distinct doublet, with one peak at 396.0 cm^−1^ and the other at 404.5 cm^−1^. The high frequency peak (404.5 cm^−1^) closely matches that of SiO_2_. The appearance of the 396.0 cm^−1^ peak is attributed to doping effects,^[^ ^29^, ^36^ ^]^ suggesting a doped‐electron concentration of 3.6 × 10^13^ cm^−2^ (equivalent of 0.033 extra electrons per unit cell).^[^ ^37^ ^]^ The $E_{2g}^{1}$ mode is sensitive to in‐plane strain but less to doping, while the *A*_1g_ mode is sensitive to doping but not significantly to strain. For the strain and doping observed here the covariance of the $E_{2g}^{1}$ and *A*_1g_ modes is negligible, and shifts in each can be taken to arise solely from strain or doping, respectively.^[^ ^36^ ^]^

The intensities of both the E(L) peak and the A(L) peak (the low‐frequency components of the $E_{2g}^{1}$ and *A*_1g_ modes, respectively) decrease with thickness (Figure 2d). This is because the light emission from the adhered bottom monolayer is damped due to absorption by the top layer(s), which becomes stronger with increasing thickness. Furthermore, as thickness increases, the top layer(s) emission becomes stronger, obscuring the signals of the bottom adhered layer. These factors lead to the diminished intensities of the E(L) and A(L) modes with increasing thickness. The E(L) mode is visible up to four layers, while the A(L) mode is only clearly visible on monolayers. Very weak signals of the A(L) mode can be identified in 2–3L flakes, as indicated by the little humps in the spectra, which are not present for SiO_2_ samples (since the intensities of the A(L) mode in 2L and 3L flakes are very weak, the fitting results are not reliable, and only the monolayer peak position is presented in Figure 2c). It is not exactly clear why the intensities of the two modes attenuate differently. We suspect this might be related to the nature of the in‐plane and out‐of‐plane vibrations of the E(L) mode and the A(L) mode, respectively (see Supporting Information for more detailed discussion).

### 2.3 ULF Raman: Shear and Breathing Modes

**Figure 3:**
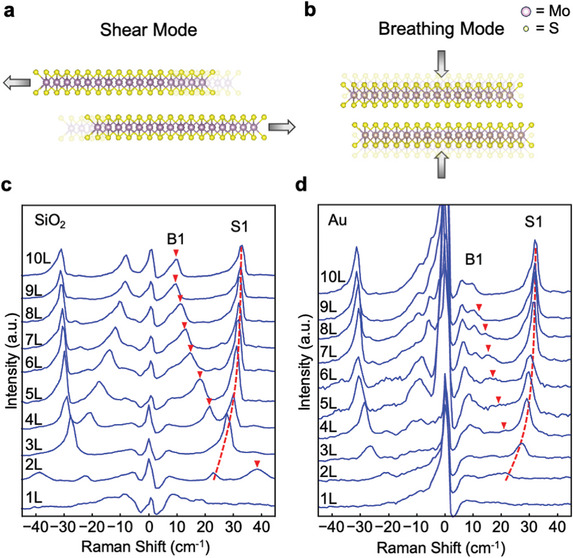
ULF Raman spectra of MoS_2_. (a,b) Schematics of the vibrations of shear and breathing modes, respectively. (c,d) Measured ULF Raman spectra of 1–10L MoS_2_ on c) SiO_2_ and d) 10 nm Au. The dashed lines serve as a guide to the eye for the first‐order shear (S1) modes. Triangles mark the locations of the first‐order breathing (B1) modes, which are notably suppressed on Au.

**Figure** **3** shows the ULF Raman spectra of 1–10L MoS_2_ on 10 nm Au and SiO_2_ substrates. The weak interlayer interactions within 2D materials often manifest as ULF Raman modes with frequencies below 100 cm^−1^.^[^ ^25^, ^26^, ^27^, ^28^ ^]^ Such modes normally consist of two types of vibrations: the shear modes for which individual layers move relative to each other parallel to the layer plane (Figure 3a) and the breathing modes for which the layers move perpendicularly to the layer plane (Figure 3b).

For MoS_2_ on SiO_2_, both the first‐order shear modes (S1) and the first‐order breathing modes (B1) are strong and clearly visible (Figure 3c). The S1 mode upshifts while the B1 mode downshifts with the number of layers, consistent with the literature.^[^ ^25^, ^26^, ^27^ ^]^ The spectra on Au are distinctly different. The S1 modes on Au are clearly visible, but the B1 modes (marked by triangles) are significantly suppressed (Figure 3d). The exact mechanism for the suppression of the B1 modes is not clear. A similar phenomenon is attributed to the quasi‐covalent bonding between MoS_2_ and Au.^[^ ^38^ ^]^ However, this factor alone cannot explain why the suppression is observed in crystals up to 10 layers and is selective for the breathing mode. We find that the intensity of the B1 modes varies with Au thickness and appears to be stronger on 2 nm and TS Au than on other Au substrates (Figure S3, Supporting Information). This suggests that there are other mechanisms contributing to the quenching of the B1 modes.

**Figure 4:**
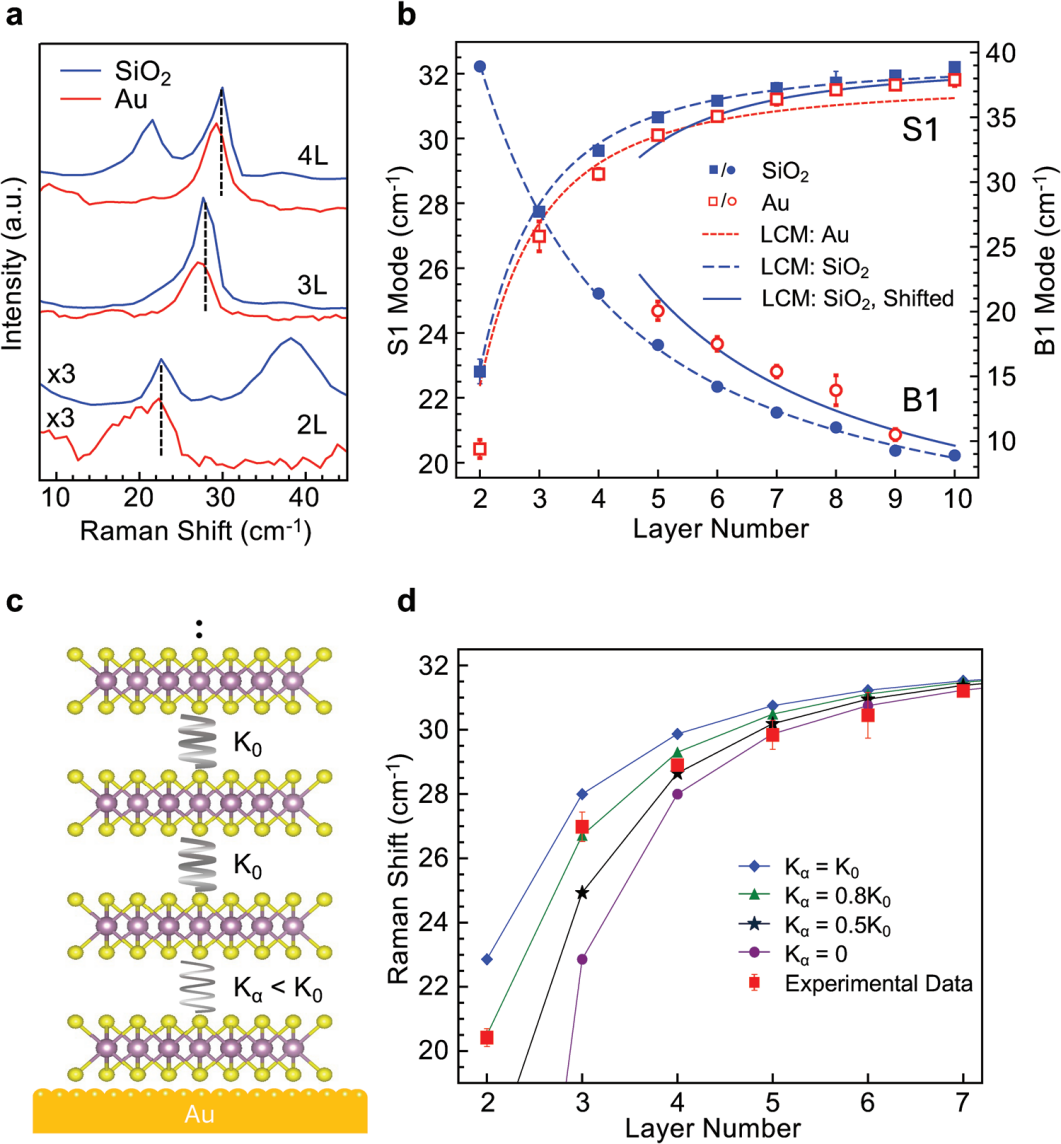
Layer‐dependent ULF Raman modes of MoS_2_ and LCM fitting. a) ULF Stokes Raman spectra of 2–4L MoS_2_ on SiO_2_ and 10 nm Au substrates, showing the downshift of the S1 modes on Au with respect to those on SiO_2_. Dotted lines mark the peaks of MoS_2_ on SiO_2_. b) S1 and B1 modes of MoS_2_ on SiO_2_ (filled symbols) and 10 nm Au (unfilled symbols) substrates. Dashed blue curves: LCM fitting of the S1 and B1 experimental data on SiO_2_ substrates. Dotted red curve: LCM fitting of the S1 experimental data on Au substrates, which overestimates the frequencies of thin crystals (< 6 layers) and underestimates those of thicker ones (> 6 layers). Solid blue curves: dashed blue curves shifted to the right by one layer, well matching the data points of ≥ 5L MoS_2_ on Au. c) Schematic illustrating a modified linear chain model (mLCM) in which the force constant *K*_α_ at the first MoS_2_‐MoS_2_ interface is variable, while the force constants at other interfaces are kept at *K*_0_, same as that of a pristine MoS_2_. d) Solid lines with symbols: S1 modes calculated by mLCM of varying *K*_α_. Comparing the experimental data on Au with the calculated frequencies provides the reduced force constants *K*_α_ at the first interface for crystals of different thicknesses. The *K*_α_ is ≈0.8*K*_0_ for bilayer and trilayer MoS_2_ crystals, ≈0.5*K*_0_ for tetralayer, and nearly zero for crystals thicker than 5 layers, indicating the bottom adhered layer is almost fully decoupled from the top layers.

The S1 modes and B1 modes of MoS_2_ on Au shift with respect to those on SiO_2_. To see this more clearly, we plot the Stokes Raman spectra of 2–4L MoS_2_ on Au and SiO_2_ together in **Figure** **4**a. The S1 modes on Au are downshifted with respect to those on SiO_2_. Figure 4b presents the frequencies of S1 and B1 modes of 2–10L MoS_2_ on Au and SiO_2_. The S1 frequency of bilayer MoS_2_ on Au is downshifted by 2.2 cm^−1^ compared to MoS_2_ on SiO_2_. The difference decreases with increasing MoS_2_ thickness. By contrast, the B1 mode is upshifted for MoS_2_ on Au compared to SiO_2_.

### 2.4 Linear Chain Model and Decoupling Effect

To quantify these shifts, we employ a linear chain model (LCM), which can be used to calculate the shear and breathing modes of pristine 2D crystals. The model treats each individual layer as a mass point, and the interaction between adjacent layers is represented by springs with a universal force constant. For an N‐layer MoS_2_ crystal, the model yields analytical solutions for the S1 and B1 mode frequencies, given by:^[^ ^25^, ^27^ ^]^

(1) $$\omega_{S1}=\sqrt{\frac{K_{x}}{2\mu \pi^{2}c^{2}}\left(1-\cos\left(\pi \left(\frac{N-1}{N}\right)\right)\right)}$$

(2) $$\omega_{B1}=\sqrt{\frac{K_{z}}{2\mu \pi^{2}c^{2}}\left(1-\cos\left(\frac{\pi }{N}\right)\right)}$$

where *ω*_S1/B1_ are the thickness‐dependent frequencies of the S1/B1 modes, respectively, *K*_*x*/*z*_ are the respective interlayer spring constants, *c* is the speed of light and *µ* is the mass per unit area of MoS_2._

Both the S1 and B1 modes of MoS_2_ on SiO_2_ substrates can be well fitted with the LCM model (Figure 4b, filled symbols and dashed blue curves), obtaining a value of (2.8 ± 0.1) × 10^19^ Nm^−3^ for *K_x_* and a value of (8.3 ± 0.1) × 10^19^ Nm^−3^ for *K_z_*. These agree well with the literature averages of (2.8  ±  0.1) × 10^19^ Nm^−3^ and (8.8  ±  0.1) × 10^19^ Nm^−3^ for *K_x_* and *K_z_* respectively.^[^ ^26^, ^27^, ^39^ ^]^ The reliable fit by the LCM model indicates that SiO_2_ substrates have minimal impact on the interlayer interaction of the exfoliated MoS_2_ layers, confirming that SiO_2_ is a good, weakly interacting reference substrate.

The results on Au, however, cannot be fitted with the LCM model with a universal force constant. Naïvely using this model to fit the data of MoS_2_ on Au results in an overestimation of the frequencies of thin crystals (*N* ≤ 6) while the frequencies of thicker ones (*N* > 6) are underestimated (dotted red line, Figure 4b). This implies that the force constants are not homogeneous across the interfaces of MoS_2_ on Au. The observations that MoS_2_ crystals preferentially cleave at the first interface and that the strain is mostly localized to the adhered layer indicate that the force constant at the first interface must be different from those at other interfaces. In light of this, we employ a modified LCM (mLCM) to account for the S1 mode of MoS_2_ on Au, where the force constant at the first MoS_2_‐MoS_2_ interface, denoted as *K*_α_, is variable, while those between the remaining layers, *K*_0_ (the force constant of pristine MoS_2_), are kept constant, as schematically shown in Figure 4c. The Raman frequency for an N‐layer system can then be extracted by solving for the eigenvalues of the corresponding N × N force constant matrix representing the equations of motion:^[^ ^25^ ^]^

(3) $$\omega_{i}^{2}{\bar{u}}_{i}=\frac{1}{2\pi^{2}c^{2}\mu }\bar{D}{\bar{u}}_{i}$$

where $\bar{D}$ is the force constant matrix considering only the nearest neighbor interaction and ${\bar{u}}_{i}$ is the *i^th^* phonon eigenvector with frequency *ω*_*i*_.

By calculating the frequencies of the S1 modes at varying *K*_α_ values and matching the calculated frequencies with the experimental data on Au, the *K*_α_ values of MoS_2_ crystals of various thicknesses can be deduced. *K*_α_ is found to be thickness‐dependent, which is ≈80% *K*_0_ for bilayers, ≈50% *K*_0_ for tetralayers, and ≈0% *K*_0_ for crystals thicker than five layers (Figure 4d). A reduced *K*_α_ corresponds to weakened coupling at the first interface. The striking finding is that *K*_α_ is nearly zero for crystals thicker than five layers. This suggests that the top layers are almost fully decoupled from the adhered bottom layer. In these circumstances, the system of an N‐layer crystal is equivalent to a superposition of a monolayer attached to the Au substrate and a decoupled (N − 1) layer sitting on top. Under this assumption, the S1 and B1 modes of a N‐layer (N ≥ 5) MoS_2_ on Au should closely resemble that of a free‐standing crystal of (N − 1) layers (since monolayer does not have interlayer modes). To verify this, we shift the LCM fitting curves of the S1 and B1 modes of MoS_2_ crystals on SiO_2_ to the right by one layer (solid blue curves, Figure 4b) and find that they closely match the data points on Au for MoS_2_ thicker than five layers. These phenomena are observed on all Au samples, including 2 nm, 5 nm, and commercially purchased 100 nm TS Au films (Figure S4, Supporting Information), indicating that the observed decoupling effect is universal in GAE.

### 2.5 Bubbles: Direct Evidence of Decoupling

**Figure 5:**
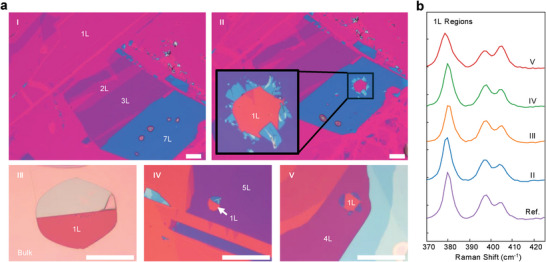
Bubbles in annealed MoS_2_ on Au. a) Optical images of MoS_2_ on a 10 nm Au substrate after annealing at 200 °C, showing the formation of bubbles in a 7‐layer flake, but not in thin flakes of 1–3L (I), and after further annealing at 400 °C, showing the burst of bubbles and the exposed monolayers in flakes thicker than three layers (II–V). All scale bars represent 25 µm. b) Raman spectra taken at the exposed regions (marked by “1L”) in (a), confirming that the exposed MoS_2_ are all monolayers. The reference spectrum of a monolayer MoS_2_ on Au, measured on a non‐heated sample, is included for comparison.

When MoS_2_ on 10 nm Au is annealed at 200 °C in the ambient environment for 5 minutes, bubbles of sizes ranging from a few microns to tens of microns are observed to form in flakes thicker than three layers, but not in thin flakes of 1–3 layers (panel I, **Figure** **5**a). When the samples are further annealed at 400 °C, the bubbles grow and some burst (panel II–V, Figure 5a). Interestingly, all the exposed surfaces are found to be monolayers (panel II–V, Figure 5a, Figure S5, Supporting Information), as confirmed by the measured Raman spectra, which show a single peak of $E_{2g}^{1}$ mode ≈380 cm^−1^ and splitting of *A*_1g_ modes, matching the spectrum of a reference monolayer on a non‐heated Au sample (Figure 5b).

Nanoscale bubbles commonly exist within the weak interfaces of heterostructures or the interfaces between 2D materials and substrates, as the result of tiny air pockets trapped at locations of defects/contaminants. When heated, the air pockets move around and coalesce, forming micron‐sized bubbles.^[^ ^40^, ^41^ ^]^ Our samples inevitably contain some defects/contaminants (the samples were prepared in the air), so it is normal to have some air pockets. Interestingly, we find micron‐sized bubbles only form in flakes thicker than three layers. No microscopic bubbles are observed in 1–3L MoS_2_ even after annealing at 400 °C (Figure 5a). This can be understood as follows. For thin flakes of two or three layers, although the interaction at the first MoS_2_‐MoS_2_ interface is weakened, it is only by a small amount (≈20%), the layers are still quite strongly bonded, so the movement of trapped air pockets is restricted, unable to form large bubbles. It is even harder for bubbles to form underneath monolayers, as monolayers are strongly pinned on the Au substrate by quasi‐covalent force. However, the first interface is significantly weakened for crystals thicker than three layers, as discussed above. As such, when the samples are heated, air pockets can escape into the weakened interface, where they can move and coalesce, forming large bubbles (the top two small bubbles in the 7L flake of panel I merge into a large bubble and burst after further annealing, as shown in panel II, Figure 5a). Since the bubbles form at the weakened interface, a monolayer surface is exposed when they burst. These observations provide direct evidence unambiguously confirming that the first MoS_2_‐MoS_2_ interface is the weakest link in the system, and for crystals thicker than three layers, the adhered bottom layer is significantly decoupled from the top layers, in excellent agreement with the conclusions of the ULF Raman investigation.

### 2.6 Mechanism of the Decoupling

Strain has been proposed as the possible cause for weakened coupling at the first MoS_2_ interface,^[^ ^3^, ^22^, ^23^ ^]^ but these are only speculations without solid experimental evidence or details of the weakening mechanism. To shed light on the effects of strain on the exfoliation, we conduct DFT simulations on the energy cost of exfoliation. For a N‐layer crystal we compare the energetic cost of two configurations: 1) a system composed of one strained layer and a detached (N − 1) layer crystal free of strain; 2) the whole crystal is strained and allowed to relax. We define the exfoliation energy as

(4) $$\Delta E_{exf}=\frac{E_{N-1}^{0}+E_{1}}{A}-\frac{E_{N}}{A}$$

where $E_{N-1}^{0}\;$ is the energy of (N − 1) unstrained layers, *E*_1_ is the energy of a separate strained layer, *E*_N_ is the energy of the whole strained stack that has been allowed to relax, and *A* is the unit‐cell area of the strained layer. Negative/positive values of Δ*E*_exf_ would indicate that monolayer exfoliation is energetically preferable/unfa, vorable.

**Figure 6:**
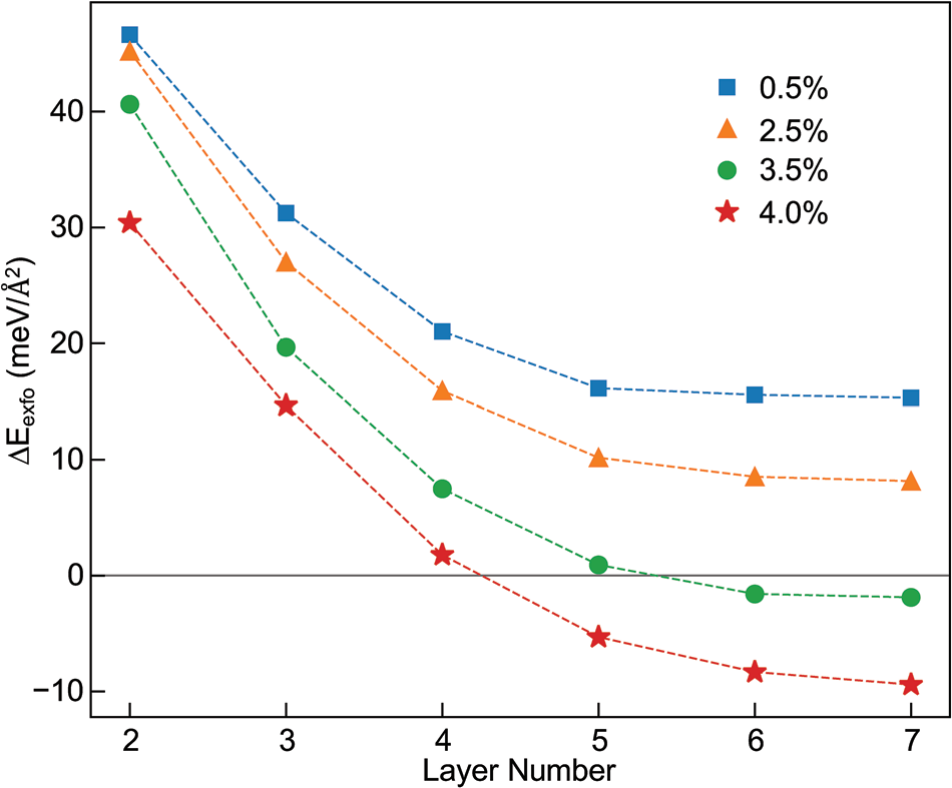
DFT simulation of the exfoliation energy of MoS_2_ crystal. Calculated exfoliation energy and its variation with strain and number of layers. Negative/positive values of Δ*E*_exf_ indicate that monolayer exfoliation is energetically preferable/unfavorable, i.e., for strains of 3.5% and larger, the monolayer is increasingly likely to exfoliate when the whole MoS_2_ crystal is thicker than five layers.

The results for various strains and layer numbers of MoS_2_ are presented in **Figure** **6**. It shows for relatively small strains, Δ*E*_exf_ is positive, hence, exfoliation is unfavorable. For strains above 3.5%, Δ*E*_exf_ crosses the zero axis and becomes negative, indicating it is energetically favorable for the top stack to be detached from the bottom strained layer. The larger the strain, the thinner the layers for which Δ*E*_exf_ crosses the zero axis, suggesting it is easier to exfoliate. The results reveal that a certain threshold strain is required to facilitate exfoliation and that the top stack tends to fully decouple from the strained bottom layer when it is thicker than a certain number of layers. Though the simulation is a simplified approximation of the actual experimental configuration, and the calculated threshold strain is larger than the strain measured experimentally, nevertheless, the simulation results provide an insightful picture of the intertwined roles of strain and thickness of MoS_2_ crystals, which are qualitatively in agreement with the experimental observations. It is worth noting that the measured strain is an averaged result. At the atomic level, the Au surface is “rough” with protrusions and troughs ranging from angstroms to nanometers.^[^ ^1^, ^29^ ^]^ At the protrusion locations, MoS_2_ makes good contact with Au, and the strain is high, which could reach or even exceed the criterion strain predicted by the simulations. At the trough regions, the contact is poorer, and the strain is lower.^[^ ^29^ ^]^ As such, the measured mean strain could be significantly lower than the maximum strain. It is the high strain at the protrusion locations that plays a central role in driving decoupling, breaking the crystal at the first interface, and initiating exfoliation. Once the exfoliation is initiated, the top crystals can be peeled off, and a large‐size monolayer can be exfoliated across the whole Au surface and even across empty holes to create suspended films.^[^ ^14^ ^]^

The simulation does not explicitly reference any specific substrates, implying that the strain‐induced decoupling effect is not limited to GAE, which also possibly plays a significant role in general mechanical exfoliations, including the scotch tape/SiO_2_ method and metal‐assisted exfoliation.

Finally, it is worth discussing the potential contribution of electrostatic force to the decoupling effect. Electrostatic repulsive force due to injected intercalation ions plays a significant role in the electrochemical exfoliation of 2D materials.^[^ ^42^ ^]^ However, in GAE, the impact of the electrostatic force due to the doped electrons by the Au substrate is minimal. As shown above, the concentration of doped electrons in the adhered layer is ∼0.033 extra electrons per unit cell. Assuming the adjacent upper layer has the same concentration of doped electrons (which is an overestimate), the coulomb repulsive force between the adhered layer and the adjacent layer is 2.3 × 10^15^ N per unit cell (the interlayer distance is taken as 0.65 nm), the equivalent of a repulsive pressure of 2.5 × 10^6^ Pa, which is only ∼0.1% of the interlayer attractive pressure due to the vdW force in MoS_2_ (2.4 GPa),^[^ ^43^ ^]^ thereby the contribution of electrostatic force to the decoupling effect is negligible. The strain induced by the gold substrate is the primary cause of the decoupling effect in GAE.

## 3 Conclusions

In summary, using MoS_2_ on Au as a model system, we establish that strain‐induced decoupling is the primary mechanism of gold‐assisted exfoliation. The gold substrate induces a biaxial strain in the adhered MoS_2_ layer, which weakens the coupling at the first interface between the adhered layer and the adjacent layer. For MoS_2_ crystals thicker than five layers, the coupling at the first MoS_2_–MoS_2_ interface is reduced almost to zero. This interface is the weakest link in the system. During exfoliation the 2D crystal preferentially cleaves at this junction, enabling the exfoliation of large‐area monolayers. These findings provide valuable insights into the development of advanced exfoliation techniques and have broad implications in diverse research and technological domains.

Previously, it was thought that the adhesion to the substrate must exceed the interlayer vdW force within the 2D crystal to achieve high‐yield exfoliation.^[^ ^1^, ^2^ ^]^ The findings in this study suggest that this stringent requirement may not be necessary. Instead, the adhesion between the substrate and the 2D crystal need only surpass the weakened binding force at the first interface, which can be considerably weaker than the interlayer vdW force when strain is present. This indicates that even substrates with relatively weak adhesion to 2D materials can facilitate large‐area exfoliation, provided sufficient strain is induced in the adhered layer to effectively weaken the interfacial coupling. These findings highlight the potential of substrate engineering, particularly the enhancement of strain in the adhered layer, as a promising strategy to extend high‐yield exfoliation techniques to a broader range of substrates and 2D materials, including dielectric substrates and 2D materials that are currently beyond the scope of gold substrates.

Interfaces play an important role in many physical and chemical phenomena (e.g., electrical and thermal conductivity, tunneling effects, charge transfer, and Schottky barriers). As such, the discovery of significant weakening at the first interface of 2D crystals on gold substrates, which may also occur on other strain‐inducing substrates, has broad implications across diverse disciplines and technologies, such as nanoelectrodes, field‐effect transistors, and photodiodes.

## 4 Experimental Section

### Sample Preparation

All thin‐film Au substrates were deposited on 100 nm SiO_2_/Si substrates with a 1 nm Ti adhesion layer (except the samples of Figure 2, which were deposited on 300 nm SiO_2_/Si substrates with a 3 nm Cr adhesion layer. The SiO_2_/Si substrates and adhesion layer had no impact on exfoliation and the results). Prior to exfoliation, SiO_2_/Si substrates were subsequently sonicated in acetone, isopropyl alcohol, and deionized water. This was followed by plasma cleaning with a 25%O_2_/75%Ar gas mixture or pure O_2_. Reference samples were prepared by bringing a MoS_2_ (HQ Graphene or Manchester Nanomaterials Ltd.) loaded tape into contact with the freshly cleaned and plasma‐activated SiO_2_ surface. The samples used for ULF Raman were not heated to avoid introducing strain. For Au samples, bulk MoS_2_ was first cleaved with tape. The freshly cleaved crystal surface was then brought into contact with a freshly prepared Au film deposited onto the cleaned SiO_2_/Si substrate with an adhesion layer through magnetron sputtering. The Au (Birmingham Metal Ltd. or Micro to Nano Ltd.) and Ti (Testbourne Ltd. or Micro to Nano Ltd.) targets used were of >99.99% purity and the Cr (Micro to Nano Ltd.) was of > 99.95%. For all depositions, the process chamber pressure was better than 9 × 10^−9^ Torr. For template‐stripped samples, pre‐prepared chips of 100 nm Au(111) (Platypus Technologies) were peeled from the protective silicon wafer, exposing a fresh, clean Au surface onto which the crystal was exfoliated. In all cases, exfoliation was carried out immediately after surfaces were exposed to air to avoid contamination.

### Raman Measurements

ULF Raman measurements were carried out using a home‐built ULF Raman apparatus operating at 532 nm in a backscattering configuration. The system was equipped with three BraggGate notch filters (BNF), allowing for ULF measurements down to 7 cm^−1^. A 100 × objective with N.A. of 0.9 was used for all measurements and the spot size was ≈0.5 µm in diameter. Laser power was kept below 1.5 mW (measured at the sample) to avoid heating. Spectra were measured with an Acton SpectraPro SP‐2300 spectrometer (Princeton Instruments) equipped with an iDus 416A CCD (Andor) and an 1800 grooves per mm grating at 500 nm blaze. The spectral resolution of the system was 2.1 cm^−1^, estimated from the full width at half maximum of the Rayleigh line, and each pixel of the CCD spans 0.8 cm^−1^. For analysis, all peaks were fitted with a Lorentzian function, and background subtraction was carried out as necessary. The positional error of the peaks was taken to be the error of the fit. The high‐frequency Raman spectra were obtained using a WITec alpha300 R Raman spectrometer (Oxford Instruments) with 532 nm excitation wavelength and 1800 grooves per mm grating. The laser was focused through a 100 × objective (Zeiss EC Epiplan‐Neofluar) with power set to 0.5 mW to avoid damaging the samples.

### AFM Measurements

AFM measurements were taken on an Asylum MFP‐3D infinity machine in tapping mode with a 25 nm PPP‐EFM tip (Nanosensors). Since the substrate interaction can introduce a large degree of variance in measured thicknesses, where possible, flakes were measured with respect to a monolayer to minimize uncertainty.

### DFT Simulations

The plane wave pseudopotential suite QUANTUM ESPRESSO was employed to perform fully self‐consistent DFT‐based electronic structure calculations by solving the standard Kohn‐Sham equations.^[^ ^44^, ^45^ ^]^ Ultrasoft pseudopotentials from the PS library were used for Mo and S atoms.^[^ ^46^ ^]^ Kinetic‐energy cut‐offs were fixed to 80 Ry for electronic wave functions after performing rigorous convergence tests. The electronic exchange–correlation was treated under the generalized gradient approximation parametrized by Perdew‐Burke‐Enzerhof functional.^[^ ^47^ ^]^ The Monkhorst‐Pack scheme was adopted to sample the Brillouin zone in k‐space with a 12 × 12 × 1 grid.^[^ ^48^ ^]^ Geometry optimization was performed using the Broyden–Fletcher–Goldfarb–Shanno scheme.^[^ ^49^ ^]^ The Convergence threshold of 10^−10^ and 10^−5^ were used on total energy (a.u.) and forces (a.u.) respectively for ionic minimization. The van der Waals interactions between layers were included in the calculation by incorporating the DFT‐D3 method.^[^ ^50^ ^]^ The multi‐layered MoS_2_ systems were prepared with the software VESTA.^[^ ^51^ ^]^

## Conflict of Interest

The authors declare no conflict of interest.

## Supporting information



Supporting Information

## Data Availability

The data that support the findings of this study are available from the corresponding author upon reasonable request.
