# Supplementary material for: Strain‐Induced Decoupling Drives Gold‐Assisted Exfoliation of Large‐Area Monolayer 2D Crystals
Source: Adv Mater. 2025 Feb 19;37(14):2419184. doi: 10.1002/adma.202419184 (PMC11983244; doi:10.1002/adma.202419184)
Supplement: Supplementary file 1 — Supporting Information [file ADMA-37-2419184-s001.pdf]

ADVANCED MATERIALS

Supporting Information

for *Adv. Mater.*, DOI 10.1002/adma.202419184

# Strain-Induced Decoupling Drives Gold-Assisted Exfoliation of Large-Area Monolayer 2D Crystals

*Jakob Ziewer*, Abyay Ghosh, Michaela Hanušová, Luka Pirker, Otakar Frank, Matěj Velický, Myrta Grüning and Fumin Huang**

# Supporting Information

# Strain-Induced Decoupling Drives Gold-Assisted Exfoliation of Large-Area Monolayer 2D Crystals

*Jakob Ziewer*[†], *Abyay Ghosh*[†], *Michaela Hanušová*[‡§], *Luka Pirker*[‡], *Otakar Frank*[‡], *Matěj Velický*[‡], *Myrta Grüning*[†], *and Fumin Huang*[†]

[†] Centre for Quantum Materials and Technologies, School of Mathematics and Physics, Queen's University Belfast, University Road, Belfast, BT7 1NN, United Kingdom

[‡] J. Heyrovský Institute of Physical Chemistry, Czech Academy of Sciences, Dolejškova 2155/3, Prague, 18223, Czech Republic

§ Faculty of Chemical Engineering, University of Chemistry and Technology, Prague, Technická 5, 166 28 Prague 6, Czech Republic

1. **Figure S1: Optical images of $MoS_2$ films exfoliated on Au substrates**

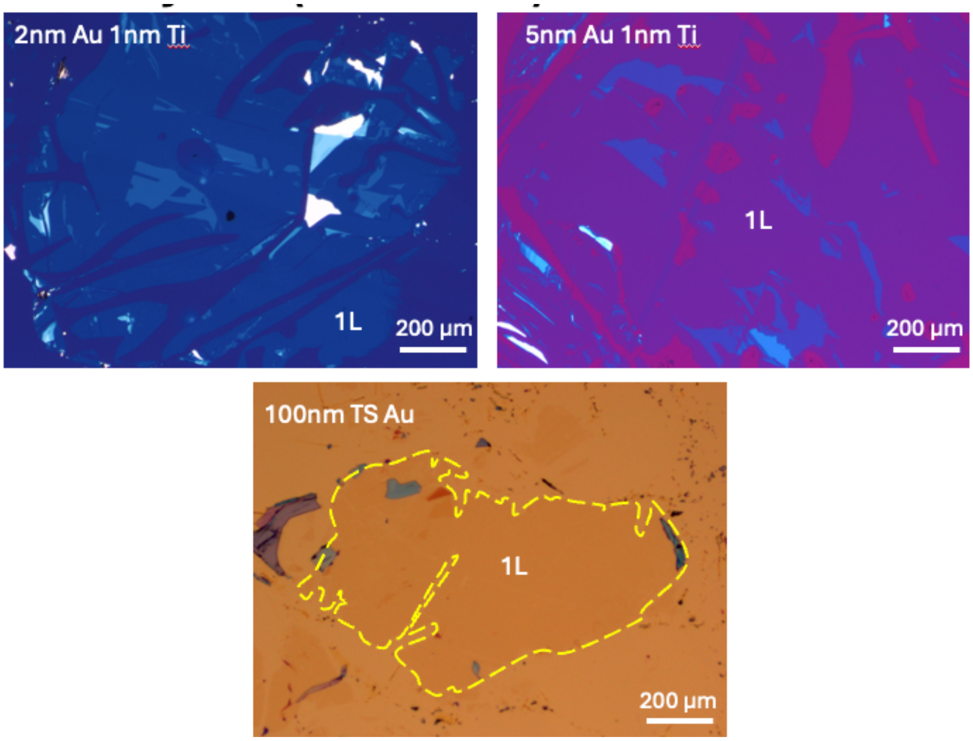


**Figure S1.** Optical images of $MoS_2$ films exfoliated on thin Au films of 2 nm, 5 nm, and template-stripped 100 nm Au film. The exfoliated crystals are predominantly monolayers (marked by "1L") exceeding 1 mm in the lateral dimension. Dashed line is drawn to mark the boundary of a monolayer on TS Au due to the relatively low optical contrast on the TS Au substrate.

## 2. Figure S2: AFM characterization of $MoS_2$ thickness

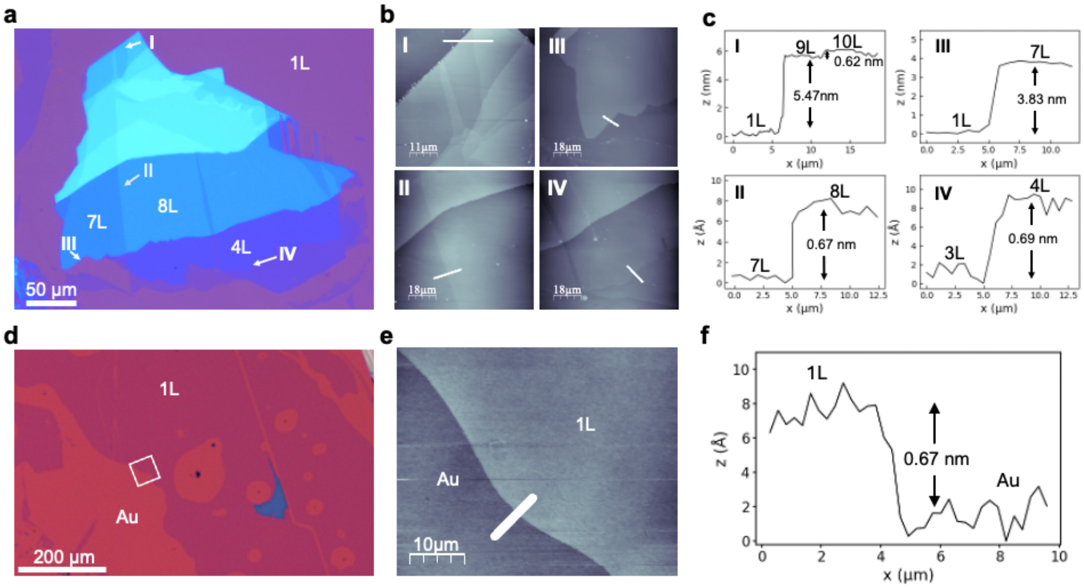


**Figure S2.** Optical image and AFM characterization of a multilayer $MoS_2$ flake exfoliated on a 10 nm Au film. (a) Optical image of a multilayer flake, which is sitting on top of a large monolayer. (b) Examples of AFM images of the marked areas in (a). (c) Topography height profiles corresponding to the marked lines in (b). (d) Optical image of a monolayer $MoS_2$ on Au. (e) AFM measurement of the marked region in (d). (f) Topography height profile corresponding to the marked line in (e).

### 3. Figure S3: ULF Raman spectra of few-layer $MoS_2$ films on various Au substrates

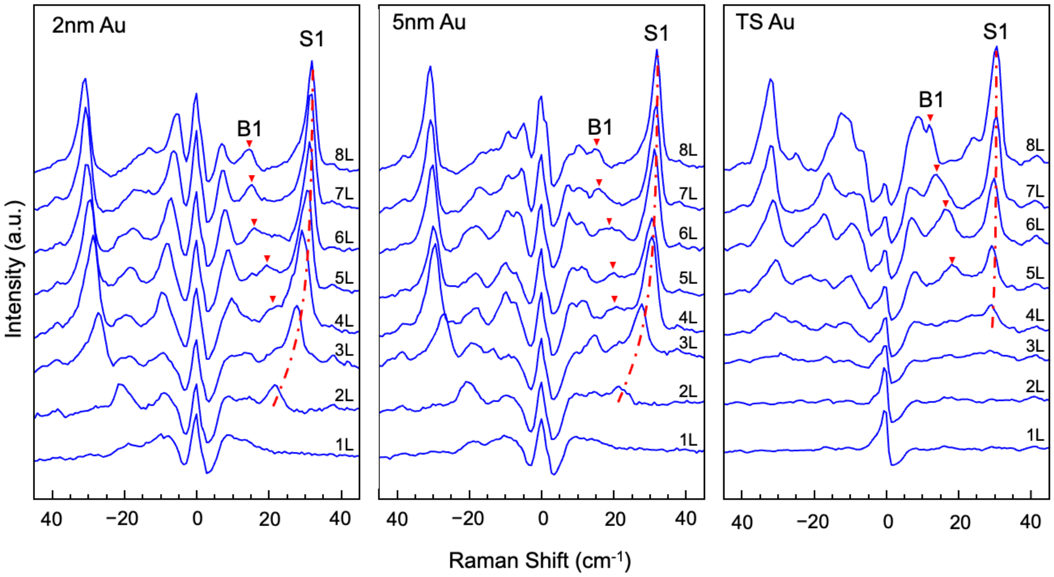


**Figure S3.** ULF Raman spectra of $MoS_2$ layers exfoliated on 2 nm, 5 nm, and template-stripped (TS) 100 nm Au films. The intensities of the first-order breathing modes (B1, marked by triangles) are generally weaker than those of the first-order shear modes (S1). The B1 modes on 2 nm Au and TS Au are stronger than those on 5 nm Au, while the S1 modes on TS Au are weaker than those on 2 nm and 5 nm Au. These suggest that the intensities of ULF Raman modes are impacted by the Au substrates.

## 4. Figure S4: Decoupling effects of $MoS_2$ on various Au substrates

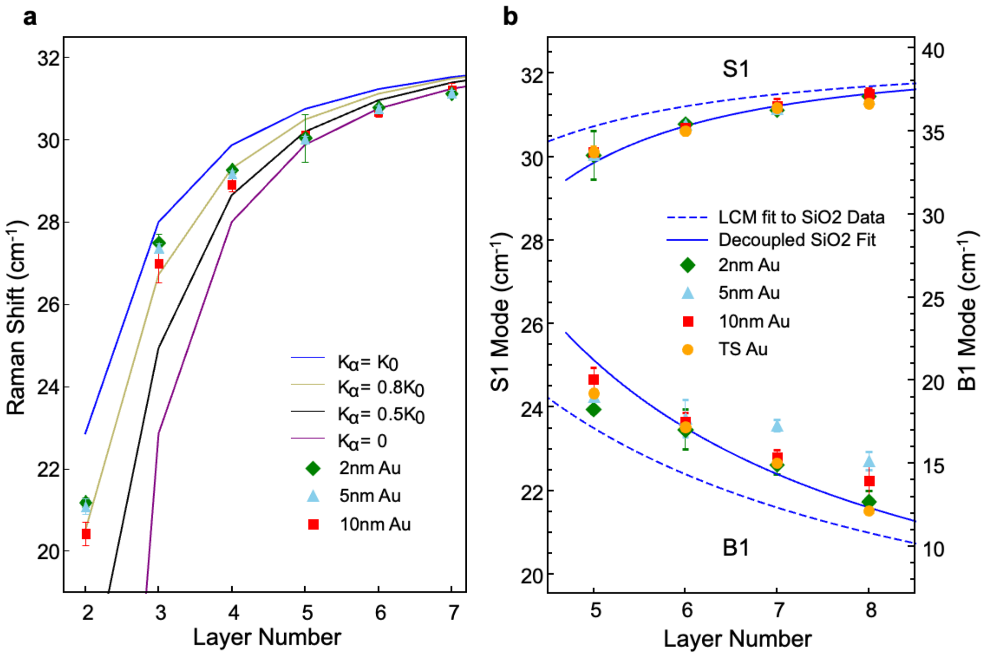


**Figure S4.** Decoupling effects of $MoS_2$ on various Au substrates. (a) Solid curves: calculated S1 modes of $MoS_2$ using a modified linear chain model (mLCM) with different values of the force constant $K_\alpha$ at the first $MoS_2$-$MoS_2$ interface. Symbols: measured S1 modes on various Au substrates, which are all below the $K_\alpha = K_0$ curve, indicating that the coupling at the first interface is weakened in all cases. $K_\alpha$ is smaller for thicker layers, suggesting the decoupling effect is more pronounced on thicker crystals. (b) Dashed lines are the fitting curves of the S1 and B1 modes of $MoS_2$ on $SiO_2$ using the standard linear chain model (LCM). Solid lines are the dashed lines shifted to the right by one layer, which match the experimental data of $MoS_2$ (thicker than 5 layers) on various Au substrates quite well. The B1 modes of $MoS_2$ on 5 nm Au have larger uncertainties due to much weaker signals. The good match between the shifted $SiO_2$ LCM fitting curves and the experimental data of $N \geq 5$ layer $MoS_2$ on Au implies that the bottom adhered layer is decoupled from the top (N-1) layers, so the system is equivalent to the superposition of a strained adhered bottom layer (which does not have interlayer vibration modes) and a stack of unstrained (N-1) top layers. The decoupling effect was observed for $MoS_2$ on all Au substrates, suggesting that it is a universal phenomenon in gold-assisted exfoliation.

5. **Figure S5: Optical images of burst bubbles**

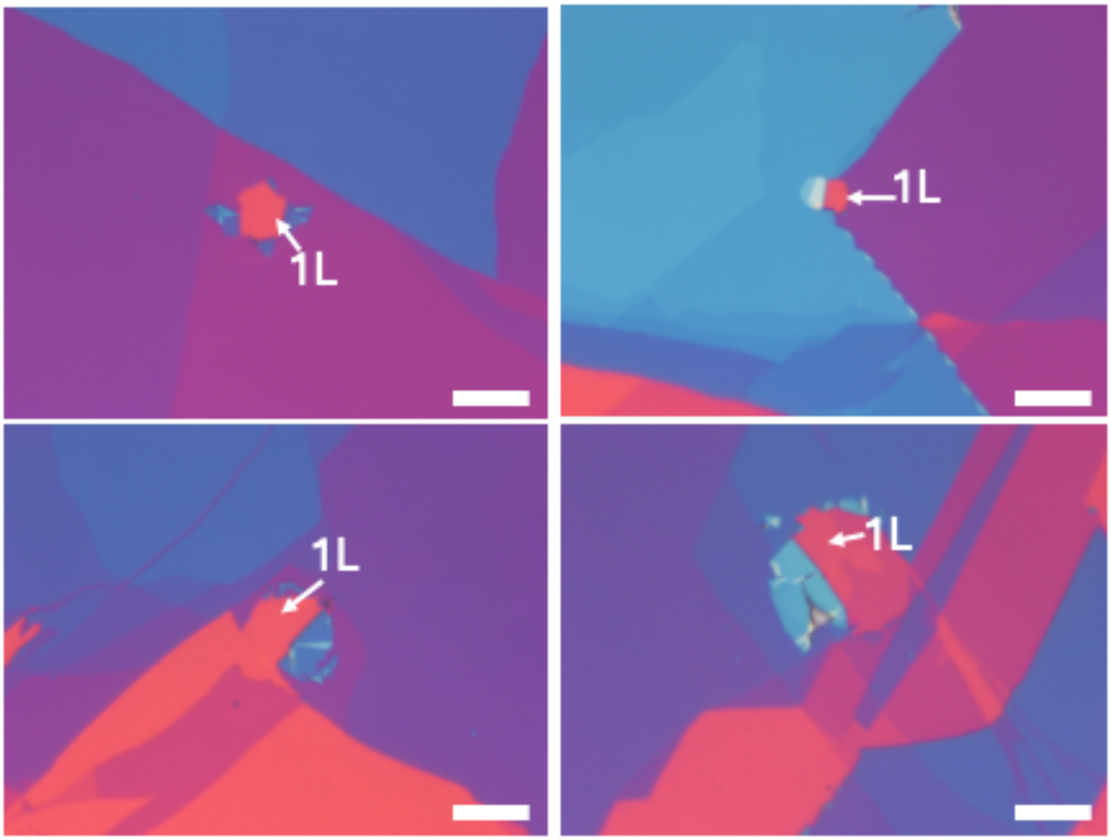


**Figure S5.** Optical images of burst bubbles in thick $MoS_2$ films exfoliated on a 10 nm Au substrate, after annealed at 400 °C. The exposed surfaces are all monolayers (marked by '1L'). Scale bars represent 10 μm.

6. **Discussions on the intensity attenuation of the E(L) and A(L) mode**

The signals of both the E(L) and A(L) modes are attenuated by the top layers, due to multiple factors, including the blockage and absorption of top layers as well as the increased signals of the top layers with thickness. These have been discussed in the manuscript. However, the A(L) mode attenuates more rapidly than the E(L) mode. We suspect this is potentially related to the in-plane and the out-of-plane vibrations of the E mode and the A mode, respectively. The out-of-plane (in-plane) mode can be approximately modelled as a vertical (horizontal) dipole oscillating

perpendicular (parallel) to the plane of 2D crystals. The radiation power of an electric dipole is mostly concentrated at large angles away from the dipole orientation, following the $(\sin\theta)^2$ law, where $\theta$ is the angle between the radiation direction and the dipole orientation. For a vertical dipole, this means the emitted light mostly propagates at a large incident angle with respect to the normal of the plane of 2D material, so more light will be reflected back and absorbed (for inclined incidence, the travel distance in the top layer(s) is longer) by the top layer(s). Therefore less signal will be transmitted through the top layer(s). For a horizontal dipole, most light is emitted in the direction normal to the plane of the 2D material, so less light will be reflected and absorbed by the top layers, and hence more light can transmit through. This will result in a stronger attenuation of the A(L) mode. In addition, the emission of a vertical dipole contains significant portion of p-polarized light, while the emission of a horizontal dipole mostly contains s-polarized light, with respect to the plane of the 2D material. For a vertical dipole, some part of its emission could transfer energy to the top layer(s) through the excitation of surface plasmons (which can only be excited with p-polarized light) in the top layer(s). This factor could also contribute to the attenuation of the A(L) mode.
